# Visual Learning in Electrocardiography Training for Medical Residents: Comparative Intervention Study

**DOI:** 10.2196/73328

**Published:** 2025-06-13

**Authors:** Heng-You Sung, Feng-Ching Liao, Shu-I Lin, Han-En Cheng, Chun-Wei Lee

**Affiliations:** 1 Cardiovascular Division, Department of Internal Medicine, Mackay Memorial Hospital, No. 92, Sec. 2, Zhongshan N. Rd., Zhongshan Dist., Taipei, 251, Taiwan, 886 975835860; 2 School of Pharmacy, National Yang Ming Chiao Tung University, Taipei, Taiwan

**Keywords:** flipped classroom, electrocardiogram learning, lecture, postgraduate education, junior residents

## Abstract

**Background:**

Although electrocardiogram (ECG) interpretation training begins early in medical school, achieving accuracy in interpretation of 12-lead ECG remains a persistent challenge. We conducted a pilot educational program to compare the effectiveness of a conventional didactic lecture, self-drawing, and self-drawing following a flipped classroom (SDFC) approach.

**Objectives:**

This study aimed to evaluate the effectiveness of three instructional strategies—traditional didactic lecture, self-drawing, and SDFC approach—in improving ECG interpretation skills among first-year postgraduate (PGY-I) medical residents.

**Methods:**

This study was conducted among postgraduate-year PGY-I residents at MacKay Memorial Hospital over 3 years. The study enrolled 76 PGY-I residents, who were randomized into three groups: conventional control (group 1), self-drawing (group 2), and SDFC (group 3). All participants were provided with the same learning material and didactic lectures. Knowledge evaluation was performed using pre- and posttests, which were administered using questionnaires.

**Results:**

The groups involving self-drawing, both combined with and without a flipped classroom approach, demonstrated better performance on the written summative examination. These findings highlight the benefits of self-drawing in integrating theoretical knowledge with practical approaches to ECG interpretation.

**Conclusion:**

Our study demonstrated promising effects of self-drawing on the recognition of ECG patterns, which could address the inadequacies of traditional classroom teaching. It can be incorporated into routine teaching after validation in a larger cohort.

## Introduction

Electrocardiogram (ECG) interpretation remains one of the most important diagnostic tools in health care for screening, early diagnosis, and treatment of cardiovascular diseases such as arrythmias and acute coronary syndrome [1]. ECG interpretation is a cognitive skill that requires considerable time and effort to master [2].

Inaccurate ECG interpretation can lead to missed critical diagnoses, resulting to failure in providing life-saving treatments (eg, complete atrioventricular block, ventricular arrhythmias, and acute coronary syndrome) or unnecessary medical interventions [3,4]. Bogun et al [3] reported that misdiagnosing atrial fibrillation in 4% of patients led to inappropriate treatments such as the unwarranted use of anticoagulant or antiarrhythmic therapies.

Teaching ECG interpretation poses significant challenges due to its complexity, often leading to reluctance among students while engaging with the subject. Although a variety of pedagogical materials (eg, textbooks, research articles, quizzes, videos) are available, and teaching methods vary across medical schools and countries (eg, self-directed learning, workshop-based training, lecture-based instruction), the most effective approach remains unclear [4-7]. It is important to note that self-directed learning refers to student-led engagement with content, while self-drawing is a structured activity aimed at enhancing visual-spatial learning through active reconstruction of ECG patterns. Flipped classroom, another active learning strategy, involves learners reviewing instructional content (eg, videos or readings) before class, allowing in-class time for discussion, problem-solving, and interactive application.

Research indicates that self-directed learning correlates with lower interpretation competence, whereas summative assessment is associated with improved interpretation competence compared to formative assessment [4]. Studies have suggested that self-drawing enhances memory retention, particularly by reinforcing the characteristic waveform patterns of specific cardiac conditions [5]. Additionally, flipped classroom models have been reported to increase student engagement and facilitate active learning in ECG education [6-8].

This study aimed to evaluate the effectiveness of different ECG teaching strategies, including lecture-based learning (LBL) alone, LBL combined with self-drawing, and LBL combined with self-drawing and a flipped classroom model. By assessing the impact of these approaches, this study aimed to identify effective pedagogical strategies for improving ECG interpretation skills among medical trainees.

## Methods

### Study Design and Participants

This retrospective study was conducted between September 2020 and June 2023 and included three consecutive cohorts of first-year postgraduate residents at MacKay Memorial Hospital. Participants were divided into three instructional groups based on their training year, with each group drawn from a distinct cohort:

- Group 1 (LBL only) included residents from the 2020‐2021 academic year (September 2020 to June 2021)
- Group 2 (lecture + self-drawing) from the 2021‐2022 academic year (July 2021 to June 2022), and
- Group 3 (lecture + self-drawing + flipped classroom) from the 2022‐2023 academic year (July 2022 to June 2023)

**Figure 1.**
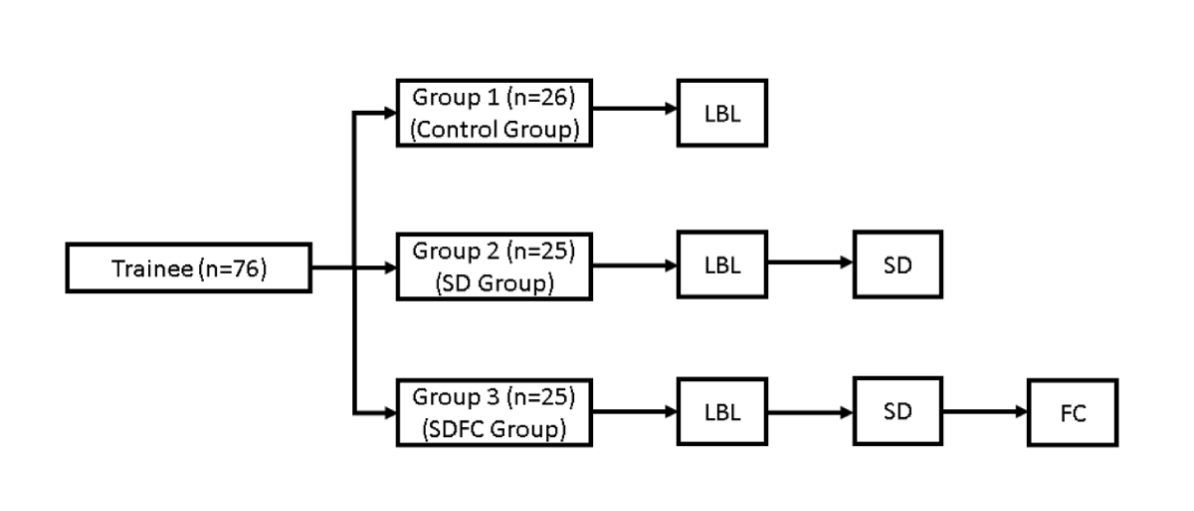
Schematic demonstration of the process of teaching activities. Group 1: Control group: traditional lecture-based learning (LBL) method; Group 2: traditional LBL and self-drawing (SD) method; Group 3: traditional LBL+SD+flipped classroom (SDFC) method.

Participants were categorized into three groups: group 1 (control group) underwent conventional LBL; group 2 (self- drawing; SD group) received the same LBL but incorporated self-drawing exercises; and group 3 (self- drawing after flipped classroom; SDFC group) engaged in LBL and the same self-drawing exercises as group 2, followed by a flipped classroom approach (Figure 1).

All participants completed a pretest at the beginning of their cardiovascular ward rotation and a posttest on the final day. The interval between the pre- and posttest was approximately 2 weeks, corresponding to the duration of each participant’s cardiovascular ward rotation.

### Conventional LBL Approach

The control group (ie, group 1) was provided preclass learning materials 3 weeks before the teaching session. These materials included textbook chapters, supplementary electrocardiogram resources, online materials, and ECG exercises. During the teaching session, instruction was delivered in a traditional LBL format, consisting of a 120-minute lecture followed by a 15-minute question-and-answer session. After the session, students were given access to the lecture slides for self-directed learning.

### Self-Drawing Method

In group 2, students engaged in self-drawing exercises to enhance cognitive processing of ECG patterns. The instructor first illustrated a characteristic ECG pattern for a specific disease, highlighting key features. After the explanation, students were instructed to replicate the ECG from memory without assistance after a 10-minute interval. Upon completion, students’ drawings were compared against the standard reference ECG, and differences were discussed to reinforce understanding.

The ECG drawings were required to include the following key elements: (1) rate and rhythm of the ECG; (2) identification of P waves, QRS complexes, T waves, PR interval, QT interval, and QRS duration; (3) characteristic ECG findings for the specific disease being studied.

In group 3, the self-drawing component was introduced following the flipped classroom session.

### Flipped Classroom Method

The flipped classroom approach in group 3 consisted of two phases:

- Preclass preparation: (1) Instructor preparation: Teachers underwent microteaching training and developed microvideo lessons, each featuring a real clinical case with guided learning instructions; (2) Student preparation: Three weeks before the teaching session (ie, immediately after the pretest), students were informed of the course structure and FC requirements. They were provided with microlessons and assigned reading materials, including textbook sections, lecture slides, and supplementary ECG resources. One day before their cardiovascular ward rotation, students submitted questions, which were compiled and reviewed by instructors.

- Classroom phase: Each 70-minute session was divided into three stages: (1) Stage 1 (20 minutes): Instructors addressed students’ presubmitted questions, clarifying challenging concepts; (2) Stage 2 (20 minutes): An interactive discussion between students and instructors facilitated deeper engagement with ECG interpretation; (3) Stage 3 (30 minutes): A concise lecture was delivered emphasizing on the core and complex concepts.

### ECG Competency Assessment

To evaluate students’ comprehension and ability to apply acquired knowledge, all three groups completed the same standardized examination. To ensure consistency in difficulty levels, the questions in the pre- and posttests were reviewed and validated by two independent instructors.

The ECG cases used in the pre- and posttests featured the same diagnoses but were derived from different patients, ensuring similar ECG morphology while varying the sequence of questions. Each test consisted of 10 ECG interpretation questions, with each correct response awarded 1 point, yielding a maximum possible score of 10 points. The time limit for the test was 25 minutes.

### Statistical Analysis

Baseline characteristics of the three groups were analyzed using appropriate statistical tests. Continuous variables such as age and pretest scores, were expressed as mean (SD) and compared using one-way ANOVA. Gender distribution was presented as a proportion and analyzed using the *χ*^2^ test.

### Ethical Considerations

This study was reviewed and approved by the institutional review board of MacKay Memorial Hospital, Taiwan (IRB number: 24MMHIS128e) in accordance with the ethical standards of the Declaration of Helsinki. We prioritized the rights and welfare of our participants. For secondary analyses using existing data, the original consent or institutional review board approval encompasses secondary analysis without the need for additional consent. To ensure participant privacy, all data collected were either anonymized or deidentified. If data could not be fully anonymized or deidentified, we implemented robust protective measures, including data encryption, restricted access, and data use agreements, to safeguard participant information and maintain confidentiality. Participants in this research study are resident physicians, therefore, they did not receive monetary compensation for their involvement. Instead, we provided nonmonetary incentives such as access to professional development resources, educational materials, or opportunities for mentorship. This approach ensured transparency and fairness in the compensation process while acknowledging the valuable time and contributions of the participants.

### Within-Group Comparisons

To evaluate whether there was a significant improvement in scores from pretest to posttest within each group, paired, two-tailed *t* tests were conducted. The Shapiro-Wilk test was first applied to assess the normality of the data distribution. Since all groups met the normality assumption (*P*>.05), a parametric paired *t* test was deemed appropriate. The test was conducted separately for each group, with a significance level set at *α*=.05.

### Between-Group Comparisons

To determine whether the posttest scores differed significantly among the three groups, a nonparametric approach was chosen due to violations of normality assumptions. Specifically, Mann-Whitney *U* tests were performed for pairwise comparisons of posttest scores between groups. Effect sizes for each between-group comparison were calculated using the formula, *r*=z/√N, where z is the standardized test statistic from the Mann-Whitney *U* test and N is the total number of observations across the two groups. The resulting values were interpreted according to Cohen criteria (small: *r*=0.1; medium: *r*=0.3; large: *r*=0.5). To account for multiple comparisons across the three groups, a Bonferroni correction was applied, setting the significance threshold at *P*<.0167 (.05/3). The following comparisons were analyzed.

Pairwise comparisons were performed with one-tailed Mann-Whitney *U* tests**,** as the hypothesis was directional, aiming to determine whether higher posttest scores were observed in the latter group of each comparison.

While within-group differences (pretest vs posttest) were normally distributed and permitted the use of parametric paired *t* tests, the distribution of posttest scores across the three independent groups violated normality, warranting a nonparametric approach (Mann-Whitney *U* test) for between-group comparisons.

### Visualization and Interpretation

Results were visualized using bar plots, where the mean values for pre- and posttest were displayed for each group. Pretest values were represented in blue, while posttest values were depicted in red. Statistical significance was indicated directly on the plots, with *P* values reported as follows: *P*<.05 was considered statistically significant; conversely, *P*≥.05 was reported as nonsignificant (n.s.), with exact *P* values provided where relevant.

The visualization approach aimed to facilitate the interpretation of both within-group improvements and between-group differences, ensuring clarity in the presentation of statistical findings.

All statistical analyses were performed using SPSS (version 25.0; IBM Corp). Continuous variables such as age and pretest scores were presented as mean (SD) and analyzed using one-way ANOVA. Categorical variables such as gender distribution were expressed as percentages and analyzed using the *χ*^2^ test. The dataset consisted of three independent groups, each undergoing pre- and posttest assessments. Descriptive statistics including mean and SD were calculated for each group to summarize the central tendency and variability of the scores.

## Results

### Baseline Participants

A total of 76 trainees were enrolled in this study, with 26 students in group 1 (control group), 25 trainees in group 2 (SD group), and 25 students in group 3 (SDFC group). There were no significant differences in age, gender, or baseline ECG test scores among the three groups prior to training (Table 1). Specifically, the findings showed that there were no significant differences in age (*F*=0.134, *P*=.88) or pretest scores (*F*=0.024, *P*=.98) among the three groups, indicating a similar baseline distribution. The gender distribution (% of men) was also not significantly different among the groups (*χ*²=0.126, *P*=.94). These findings suggest that all three groups were well-balanced in terms of demographic characteristics before the intervention. (Table 1)

**Table 1.** Baseline characteristics of enrolled trainees.

Characteristics	Group 1 (n=26)	Group 2 (n=25)	Group 3 (n=25)	*F* test (*df*)/*χ^2^ (df)*	*P* value
Age (years), mean (SD)	26.5 (1.1)	26.4 (0.6)	26.4 (0.6)	0.134 (2,73)^a^	.88
Pretest score, mean (SD)	2.96 (1.31)	2.92 (1.44)	2.88 (1.20)	0.024 (2)^b^	.98
Gender (men), n (%)	80.8	84.0	84.0	0.126 (2)^b^	.94

a ANOVA.

b Chi-square (*χ*2) test.

### Within-Group Comparisons (Pretest vs Posttest)

**Figure 2.**
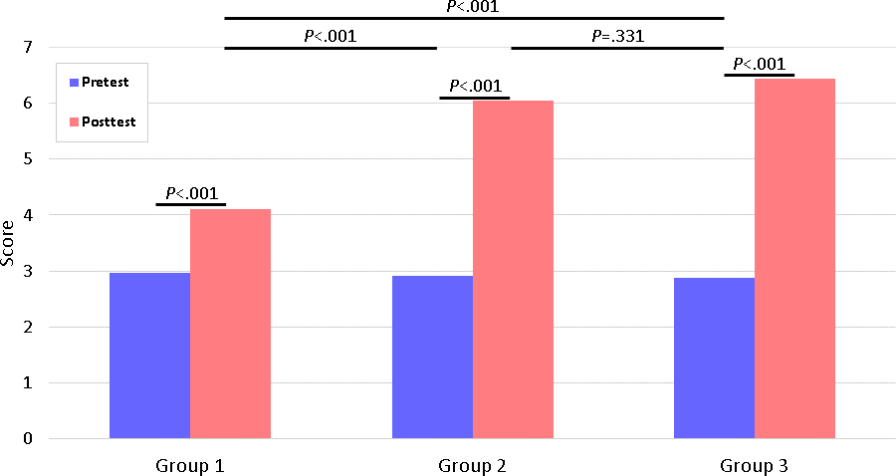
Electrocardiogram (ECG) test scores (pre- and posttest) of the three groups. Bonferroni-corrected significance threshold is *P*<.0167.

The findings demonstrated a statistically significant improvement in posttest scores across all three groups. Group 1 exhibited a significant increase from pretest to posttest. Group 2 also showed a significant improvement, with posttest scores being higher than pretest scores. Group 3 followed a similar trend, with a significant increase from pretest to posttest. These findings suggest that all three groups benefited from the training, with group 1 exhibiting the most substantial improvement (Figure 2).

### Between-Group Comparisons (Posttest Scores)

Group 2 outperformed group 1 significantly (*U*=120.0, *z*=−3.86, *P*<.001, *r*=0.541), which remained statistically significant after applying Bonferroni correction (adjusted *α*=.0167). Group 3 also significantly outperformed group 1 (*U*=75.5, *z*=−4.70, *P*<.001 , *r*=0.658), meeting the corrected significance level. However, the difference between group 2 and group 3 was not statistically significant (*U*=263.0, *z*=−0.96, *P*=.33 , *r*=0.136), even before correction. These results indicate that while group 2 demonstrated a statistically significant advantage over group 1, the difference between group 2 and group 3 was not significant. (Figure 2).

## Discussion

### Principal Findings

The results of this study demonstrate that all three groups exhibited significant improvements in their ECG interpretation skills following training, suggesting that the instructional methods employed were effective in enhancing students’ understanding and application of ECG principles. Among these methods, self-drawing emerged as a particularly impactful learning strategy, as evidenced by the superior posttest performance of students in group 2 (SD group) and group 3 (SDFC group) compared to group 1 (control group). These statistically significant differences were further substantiated by effect size metrics, which demonstrated medium-to-large magnitude improvements in ECG interpretation performance in the intervention groups compared to the control. Statistical differences between groups remained robust after applying Bonferroni correction for multiple comparisons (adjusted *α*=.0167), confirming that the observed advantages of the self-drawing and flipped classroom interventions were not attributable to chance or multiple testing artifacts.

A key reason why self-drawing may have contributed to improved learning outcomes is its ability to engage sensorimotor processes, spatial reasoning, and active recall. Blended learning yields significantly better ECG competence and confidence among medical students compared to conventional teaching. A stepwise approach to ECG analysis combined with deliberate practice and feedback may serve as an effective complement to lectures for electrocardiography education [9]. Unlike passive learning methods such as reading textbooks, watching videos, or participating in discussions, self-drawing requires students to actively reconstruct ECG waveforms, compelling them to process the anatomical and electrophysiological principles in a highly interactive manner. Prior research suggests that hand-drawn illustrations enhance memory encoding and retrieval by reinforcing visual-spatial associations [10,11]. This effect is particularly relevant for ECG interpretation, where recognizing waveform morphology and associating it with clinical conditions is crucial. The act of manually reproducing ECG patterns may thus create stronger mental representations, leading to enhanced retention and diagnostic accuracy.

Interestingly, the findings indicate that adding a flipped classroom component to self-drawing (group 3) did not lead to statistically significant gains over self-drawing alone (group 2; *P*=.18). While flipped learning has been widely recognized for enhancing engagement and promoting deeper conceptual understanding, its benefits appear to be diminished when self-drawing is already incorporated as a core learning strategy [12-17]. A previous study revealed that students using a flipped classroom format outperformed their classmates in the ability to interpret ECGs; however, this advantage was not evident when compared to lecture-delivered content. The students were able to prioritize their time when making decisions about attendance, based on teaching modality [18]. One possible explanation is that self-drawing is already an inherently active learning process, requiring students to deconstruct, analyze, and synthesize ECG patterns in a way that other instructional formats—such as passive content review or peer discussion—may not fully replicate. A previous prospective randomized trial indicated summative assessments significantly affect midterm retention of ECG interpretation skills. More intensive teaching showed no advantage over self-directed learning in retention test performance, and the substantial performance decline over eight weeks occurred independently of overall performance levels. These findings have implications for the design of ECG teaching and assessment intervention [19]. As a result, while flipped classroom elements may provide additional instructional support, their relative impact on learning may be reduced when students have already engaged in self-directed drawing exercises.

Moreover, the self-drawing process itself may offer an engaging and enjoyable learning experience, further reinforcing knowledge retention. The act of physically sketching ECG waveforms may create a sense of personal involvement that fosters intrinsic motivation, a factor that has been shown to positively influence long-term knowledge retention [5]. Student feedback on the teaching model indicated that they found self-directed activities beneficial, reinforcing the notion that active participation enhances learning engagement.

Despite these promising findings, certain challenges and limitations should be acknowledged. First, while self-drawing appears to be a highly effective tool for ECG interpretation training, its applicability to other domains of medical education remains to be explored. Second, although efforts were made to standardize the pre- and posttest difficulty levels, individual variations in artistic ability or drawing confidence could have influenced the results. Future studies should investigate whether guidance in sketching techniques or structured drawing templates may further optimize learning outcomes. Additionally, although self-drawing with flipped classroom instruction did not show a statistically significant advantage over self-drawing alone, further research is needed to explore whether longer-term retention benefits emerge when flipped learning is combined with self-drawing over extended periods.

### Limitations

Several limitations should be acknowledged in this study. First, the improvement in ECG interpretation skills was assessed by comparing pre- and posttest scores using the same exam questions, albeit with a six-week interval between assessments. It is possible that some participants may have memorized the exam questions, potentially influencing the evaluation of their true ECG interpretation ability. Second, the posttest was conducted immediately after the teaching program, which may not fully reflect long-term retention and competency in ECG interpretation. Future studies should incorporate delayed posttests to assess whether the observed improvements persist over time.

Third, although self-drawing was found to be an effective tool for ECG training, its applicability to other areas of medical education remains to be explored. Although efforts were made to standardize learning quality by using a checklist for discussions following ECG drawing, the drawing process itself is inherently subjective. Variations in artistic ability or drawing confidence among participants may have influenced the subsequent discussions and, consequently, the learning outcomes. Future research could examine whether structured guidance in sketching techniques or the use of standardized drawing templates might further optimize learning effectiveness.

Last, although self-drawing with flipped classroom did not demonstrate a statistically significant advantage over self-drawing alone, further research is required to explore whether longer-term retention benefits emerge when flipped learning is combined with self-drawing over an extended period. Additionally, investigating the interactive dynamics between different active learning modalities may provide insights into how to better integrate self-drawing within broader medical education frameworks.

### Conclusion and Future Directions

This study highlights the effectiveness of self-drawing as a learning tool in ECG education, particularly in enhancing students’ ability to recognize and interpret waveform characteristics. The findings suggest that self-drawing offers cognitive and experiential advantages that may not be easily replicated through passive learning modalities such as textbooks, videos, or discussions. While the flipped classroom remains a valuable pedagogical approach, its benefits appear to be moderated when self-drawing is already implemented as a central learning strategy. Given these insights, future research should explore the role of self-drawing across different medical education contexts and investigate how best to integrate flipped learning techniques to maximize knowledge retention and engagement.

## Abbreviations

ECG: electrocardiogram
LBL: lecture-based learning
PGY-I: first-year postgraduate
SDFC: self-drawing following a flipped classroom
